# From multi-omics to predictive biomarker: AI in tumor microenvironment

**DOI:** 10.3389/fimmu.2024.1514977

**Published:** 2024-12-23

**Authors:** Luo Hai, Ziming Jiang, Haoxuan Zhang, Yingli Sun

**Affiliations:** ^1^ Central Laboratory, National Cancer Center/National Clinical Research Center for Cancer/Cancer Hospital & Shenzhen Hospital, Chinese Academy of Medical Sciences and Peking Union Medical College, Shenzhen, China; ^2^ Shenzhen Key Laboratory of Epigenetics and Precision Medicine for Cancers, National Cancer Center/National Clinical Research Center for Cancer/Cancer Hospital & Shenzhen Hospital, Chinese Academy of Medical Sciences and Peking Union Medical College, Shenzhen, China; ^3^ Department of Thoracic Surgery, National Cancer Center/National Clinical Research Center for Cancer/Cancer Hospital, Chinese Academy of Medical Sciences and Peking Union Medical College, Beijing, China; ^4^ Key Laboratory of Genomic and Precision Medicine, Beijing Institute of Genomics, Chinese Academy of Sciences, Beijing, China

**Keywords:** tumor cells, tumor microenvironment, metabolomics, interactions, artificial intelligence

## Abstract

In recent years, tumors have emerged as a major global health threat. An increasing number of studies indicate that the production, development, metastasis, and elimination of tumor cells are closely related to the tumor microenvironment (TME). Advances in artificial intelligence (AI) algorithms, particularly in large language models, have rapidly propelled research in the medical field. This review focuses on the current state and strategies of applying AI algorithms to tumor metabolism studies and explores expression differences between tumor cells and normal cells. The analysis is conducted from the perspectives of metabolomics and interactions within the TME, further examining the roles of various cytokines. This review describes the potential approaches through which AI algorithms can facilitate tumor metabolic studies, which offers a valuable perspective for a deeper understanding of the pathological mechanisms of tumors.

## 1 Introduction

The tumor is characterized by the uncontrolled growth of abnormal cells, invasion of adjacent parts of the body beyond normal boundaries, or metastasis to other organs, ultimately leading to death (1). According to the World Health Organization, malignant tumors have become one of the major threats to human health (2). Every year, millions of people die from cancer, with lung cancer, colorectal cancer, liver cancer, breast cancer, and cervical cancer being the most common types (3). During the development and progression of tumors, tumor cells are exposed to harsh conditions (4). To survive and sustain growth, cells must adapt to the environment. Cellular metabolic reprogramming is a mechanism by which cells promote cell proliferation and growth by altering metabolic patterns to meet energy needs. Metabolic reprogramming not only helps tumor cells resist external stress but also endows them with new functions, e.g. immune suppression and evasion (5, 6). However, it is still difficult to discover the signaling pathways and mechanisms that control the metabolic reprogramming of tumor cells and immune cells.

To explore the above challenges, research on tumor metabolism has gradually entered metabolomics. Metabolomics is the collection of metabolites, or low molecule chemicals involved in metabolism, and can directly reflect the functional readouts of biochemical reactions, providing insight into many aspects of cell physiology. Metabolomics has been applied in many aspects of tumor research, including tumor pathology discovery, biomarker discovery, and treatment efficacy evaluation (7, 8). Changes in the metabolic spectrum reflect the process of tumor occurrence and development. Another important area is the exploration of personalized treatment strategies, namely the recognition of personalized tumor biomarkers (9, 10). Moreover, tumor metabolomics analysis can integrate biomolecular information, kinetic data, and other omics data to further study the activity of metabolites within tumor cells and track deep changes in metabolic pathways (11).

With technological advancements, artificial intelligence (AI) has increasingly been applied in tumor metabolomics research. AI technology offers significant advantages in handling large-scale data, uncovering complex biological networks, and improving research accuracy. Specifically, AI can be utilized in various research stages, including data preprocessing, feature extraction, pattern recognition, and data integration. For example, deep learning algorithms can identify feature peaks in mass spectrometry data, increasing the accuracy of metabolite identification. Machine learning methods can perform integrative analysis of multi-omics data, helping to reveal intricate biological networks and pathways. By integrating AI technology, researchers have achieved a more comprehensive and in-depth understanding of tumor metabolism processes. These technologies not only increase the efficiency and accuracy of data analysis but also offer new perspectives for personalized medicine and precision therapy. As AI technology continues to advance, further breakthroughs in tumor metabolism research are anticipated, driving significant progress in biomedical research.

## 2 Tumor microenvironment

**Figure 1:**
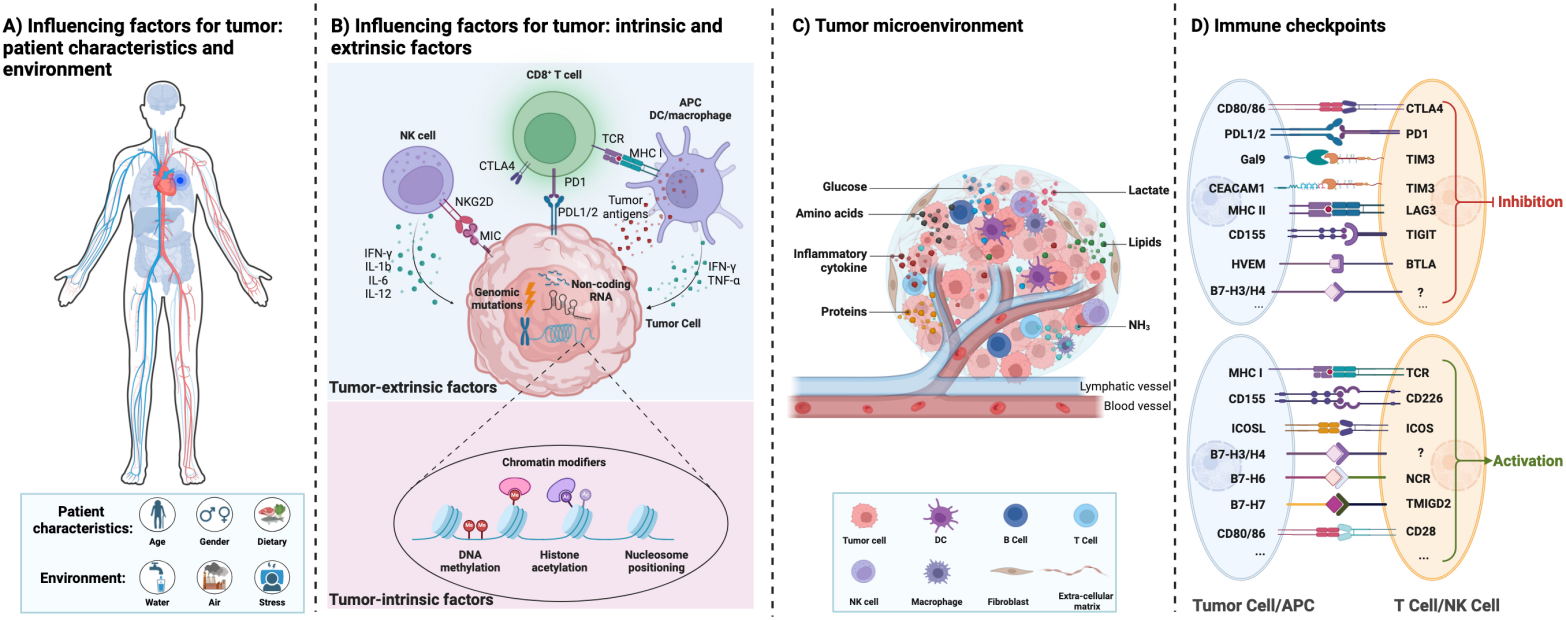
Interactions between the influencing factors of tumor, tumor microenvironment, and tumor immunity. **(A)** The different influencing factors of tumor: patient characteristics and environment. **(B)** The different influencing factors of tumor: tumor intrinsic and extrinsic factors. **(C)** The tumor microenvironment with different cell types and their interactions directly affects cancer progression. They provide tumors with abundant nutrients (including glucose, amino acids, lipids, proteins, etc.) and an immunosuppressive environment for tumor growth. **(D)** Immune checkpoint proteins between immune cells and tumor cells are summarized.

Tumors are caused by the accumulation of genetic mutations and global epigenetic changes in chromatin that regulate gene expression (12). Genetic alterations in tumor suppressor genes or oncogenes can lead to dramatic changes in gene expression leading to cancer (13). Epigenetic modifications of chromatin, including DNA methylation, histone modifications, nucleosome positioning, and non-coding RNAs, can regulate DNA access to transcription factors and other cis-regulatory elements, thereby affecting gene expression (14). Genetic and epigenetic factors complement each other to drive tumor initiation and progression. **Figures 1A, B** show a graphical representation of various patient characteristics (e.g., gender, age, and dietary), environment (e.g., water, air, and stress), tumor-intrinsic factors, and extrinsic factors affecting the cancer cells and thereby regulate the tumor microenvironment (TME) (13). Both intrinsic and extrinsic factors of tumors regulate the immune response in the TME. Genomic mutations, chromatin modifiers, and non-coding RNAs, among other intrinsic factors of cancer cells, regulate tumorigenesis, metastasis, and immunogenicity. Epigenetic modifications (such as DNA methylation and histone acetylation) regulate gene expression (15). Non-coding RNAs (including long non-coding RNAs, microRNAs, and circular non-coding RNAs) regulate gene transcription and mRNA stability (16). Other intrinsic mechanisms of cancer cells include the expression of immunosuppressive cytokines to evade anti-tumor immunity, expression of immunosuppressive molecules such as PD-L1 and PD-L2, and inhibition of antigen processing and presentation mechanisms and tumor-associated antigens (17). Extrinsic factors of cancer cells include tumor-infiltrating immune cells, fibroblasts, stromal cells, and endothelial cells (18). Extrinsic factors also include secreted factors such as cytokines, chemokines, metabolites, growth factors, and immune checkpoint molecules (19). Tumor-associated antigens presented by antigen-presenting cells such as macrophages and dendritic cells can activate CD8^+^ T cells and thereby induce effective anti-tumor immunity. However, immune checkpoint molecules expressed by cancer cells regulate the inflammatory state of tumors and suppress inflammation (**Figure 1D**) (20).

Tumor cells interact with normal cells and other factors to create TME (21–23). TME contains blood vessels, immune cells, stromal cells, fibroblasts, signaling molecules, and extracellular matrix (**Figure 1C**) (20). The TME plays an important role in the occurrence and development of tumors. Mostly, the microenvironment of early-stage tumors tends to exert anti-tumor effects, while the microenvironment of late-stage tumors tends to worsen conditions (24). Moreover, the metabolism of tumor cells is complex and heterogeneous, involving the metabolism of glucose, lactate, pyruvate, glutamine, and fatty acids (25). Tumor cells adapt to changes through remodeling in different TME, including the production of ATP bioenergy, oxygen balance, and nutrient absorption (26). Damaghi et al. (24) found through their study of ductal carcinoma *in situ* cells that the harsh TME promotes the Warburg effect through transcriptional reprogramming. Due to the potential complex metabolic interactions between the TME and tumor cells, studying the relationship between the TME and metabolism can help elucidate the remodeling mechanism of tumor tissue and the metabolic changes in tumor cells.

The metabolic characteristics and preferences of tumor cells constantly change during the development of tumors. Meanwhile, the TME is a complex and constantly changing entity whose composition varies depending on the type and location of the tumor (27). The communication between the tumor cells and TME results in specific patterns of tumor cell growth and development and has spawned various ways to help tumor cells evade immune surveillance (28). Fibroblasts regulate the production of extracellular matrix and tumor signaling molecules, promoting tumor growth, invasion, and metastasis (29). Tumor cells and specific cells of the TME avoid or inhibit immune responses by inhibiting the proliferation of helper and cytotoxic T cells or by promoting the recruitment of immune suppressive regulatory T cells (Treg) and bone marrow-derived suppressive cells (MDSC) mediated by inflammation (30). Chekulayev et al. (31) discovered the “reverse Warburg effect” when studying the crosstalk between colon cancer cells and stromal cells. Rossi et al. (32) reported that microbial metabolites can be important regulatory factors in the TME, regulating inflammation, proliferation, and cell death in a positive or negative manner. In pancreatic adenocarcinoma, stromal-associated pancreatic stellate cells secrete alanine, which provides carbon and nitrogen for tumor cell proliferation (33).

## 3 Tumor metabolism

Metabolites are extremely sensitive to internal signals and external stimuli, which means that metabolomics has the potential to become a probe of biological phenotypes, revealing what is happening inside cells. Metabolomics can systematically identify and quantify all metabolites in biological samples at high throughput, providing key information about the state of cancer that other omics technologies cannot. Metabolic perturbations can lead to characteristic metabolic phenotypes that can be used for early cancer diagnosis, surveillance, and as targets for cancer therapy. Studying tumor metabolism involves analyzing a series of biochemical reactions in tumor cells, which involve various types of reactants and their related enzymes (34–36). Therefore, studying changes in metabolism-related substances is the foundation of most tumor metabolism research.

Analytical methods to study metabolomics include the use of classical chemical analysis (37, 38), nuclear magnetic resonance (NMR) (39–41), and mass spectrometry (MS) (42–44). These methods have the advantage of high identification and quantitative accuracy when studying biochemical reactions related to tumor metabolism. However, there are also some disadvantages. For example, NMR spectroscopy requires high purity samples, which limits its applicability in analyzing the fine structure of metabolites (45). MS has been fueled by combining with highly efficient separation techniques such as gas chromatography or liquid chromatography, which improve the resolution of analytes by increasing sensitivity and specificity (46). Although these combinations allow the detection of more analytes with high sensitivity and specificity, MS-based techniques still face several challenges, such as the chromatographic separation of isomers, the elimination of exogenous contaminants, the lack of a complete reference database, and the identification of unknown metabolites (47).

Advances in single-cell sequencing, spatial transcriptomics, proteomics, and artificial intelligence have propelled tumor metabolomics into a new era. AI significantly contributes to elucidating tumor metabolism mechanisms, identifying diagnostic and prognostic biomarkers, and facilitating clinical applications. For example, Zhao et al. (48) successfully demonstrated the utility of machine learning algorithms in enhancing diagnostic accuracy for early-stage esophageal squamous cell carcinoma (ESCC). The study employed Uniform Manifold Approximation and Projection (UMAP) and Hierarchical Clustering Analysis (HCA) to categorize tissue samples into distinct groups. Orthogonal Partial Least Squares Discriminant Analysis (OPLS-DA) revealed significant metabolic differences between ESCC tumors and normal mucosa. Additionally, Random Forest analysis was utilized to identify critical metabolic biomarkers, such as glutamate, which effectively distinguished early-stage ESCC from normal tissues. Support Vector Machine (SVM) models were further applied to develop simplified metabolite panels, achieving an area under the curve (AUC) of 0.984 in serum samples. These findings illustrate that AI algorithms have become integral to nearly every aspect of tumor metabolism research (48). The emergence of advanced algorithms has significantly enhanced our understanding of tumorigenesis. For instance, Deng et al. (49) developed an explainable deep learning algorithm, DeepMSProfiler, which effectively removes batch effects by systematically excluding batch-related information through hidden layers. Building on this algorithm, the integration of metabolomics and methylation data in the study verified the associations between the PLA and UGT gene families and disease-specific metabolites. Su et al. (50) revealed the drug resistance pathway in melanoma cells through single-cell multi-omics analysis, providing a key and unique tool for addressing the signaling pathway function and metabolic changes in the adaptive development of drug resistance in tumor cells. Sun et al. (51) used spatial metabolomics to locate and analyze metabolites of different metabolic pathways in the tissues of 256 esophageal cancer patients, revealing the molecular level of tumor occurrence from metabolites to enzymes and providing a new perspective for understanding the metabolic reprogramming of tumors. Jin et al. (52) established a relationship between metabolic characteristics and oncogenic mutations of receptor tyrosine kinase by integrating metabolomics and transcriptomics, providing a basis for metabolic targeted therapy of specific tumor genotypes.

Almost all malignant tumors exhibit uncontrolled cell proliferation (53). To support cell growth, tumor cells must adaptively adjust their metabolism to meet their material and energy needs (54). Otto Warburg and colleagues first discovered that tumor cells tend to convert oxidative metabolism into fermentation metabolism (55). In normal cells, most of the pyruvate formed by glycolysis enters the tricarboxylic acid cycle and is oxidized through oxidative phosphorylation (OXPHOS) (56). In contrast, in tumor cells, most pyruvate is converted into lactate through fermentation, a phenomenon known as the “Warburg effect” (57, 58). Through competitive uptake of glucose, cancer cells gain a survival advantage over normal cells through metabolic adaptations in oxygen-limited conditions. Moreover, cancer cells need to use their nutrient inputs as cellular building blocks. Thus, cancer cells switch from OXPHOS (nutrient-consuming) to glycolysis (biomass-building) pathways to support their rapid, uncontrolled proliferation. Hu et al. (59) found that the overall metabolic status of different tumors was similar, such as the upregulation of nucleotide biosynthesis and glycolysis. However, the expression of specific metabolic pathways, such as OXPHOS, is heterogeneous among different tumors (60).

## 4 Metabolic modulation of cell-mediated immunity

With the deepening of research, researchers have discovered that tumors are essentially a metabolic disorder, in which several major metabolic pathways are altered to accommodate increased proliferation of tumor cells, and the reprogrammed metabolic pathways include glucose, amino acids, lipids, and other metabolism. The metabolic reprogramming not only meets the nutritional or energy needs of tumor cells but also affects the function of immune cells (61). Understanding the metabolic modulation of tumor immunity may provide therapeutic insights into immunotherapy resistance and facilitate the development of new strategies for tumor therapy.

### 4.1 Glycolysis metabolism

**Figure 2:**
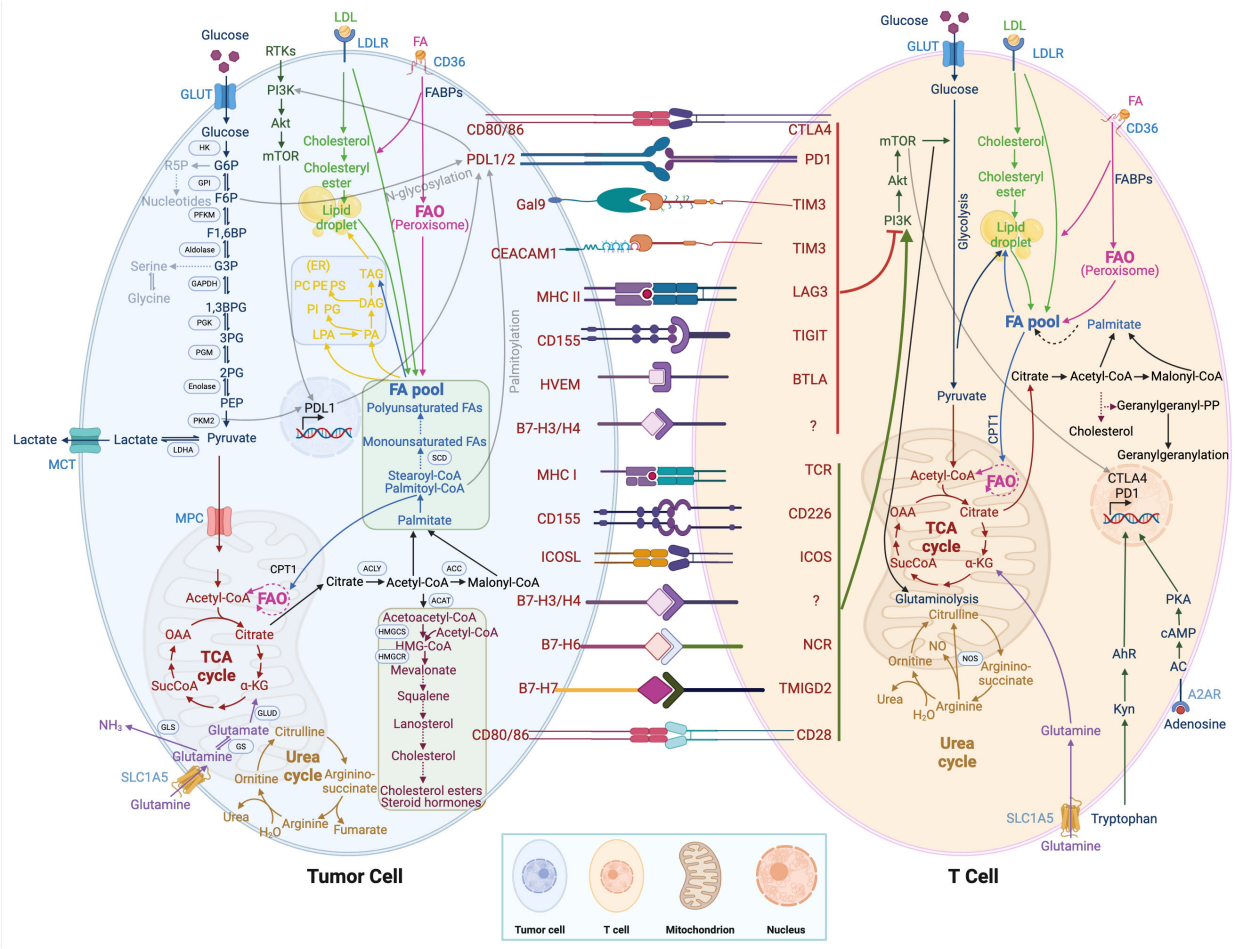
Metabolic regulation of tumor cells and immune cells. T cell immunometabolism and tumor metabolism are displayed. Crosstalk between intracellular metabolic and immune checkpoints is indicated by a transparent black line.

Glycolysis (2 mol APT/mol glucose) is a less efficient pathway for ATP production than mitochondrial OXPHOS (36 mol ATP/mol glucose). However, the rate of glycolysis is 10~100 times faster than the rate of mitochondrial TCA cycle and OXPHOS. Therefore, the amount of ATP produced by the two metabolic pathways of glucose is similar at the same time. In tumor cells, glucose is metabolized by glycolysis, producing lactate and nicotinamide adenine dinucleotides under aerobic conditions. The low-yielding but high-rate ATP production mode is more conducive to the competition of tumor cells for nutrients and meets their energy demand for rapid growth. Tumor cells take up a large amount of glucose from the environment with a strong competitive advantage, ensuring their energy supply and self-growth, depriving immune cells of glucose utilization, and inhibiting their tumor cell-killing effect. In addition, glucose metabolism including glycolysis, pentose phosphate pathway, hexosamine pathway, and glycogen synthesis are reprogrammed in tumor cells (62). As shown in **Figure 2**, pyruvate kinase M2 (PKM2), which is highly expressed in tumors and promotes the Warburg effect, is upstream of the decision point between glycolytic and oxidative metabolism. PKM2 converts phosphoenolpyruvate (PEP) into pyruvate, which can be metabolized either to lactate or acetyl-CoA (63). Moreover, PKM2 also functions as a transcriptional coactivator, PKM2 interacts with HIF-1α in the nucleus and binds to the PD-L1 promoter region, enhancing the expression of PD-L1 in tumor cells (64). The interaction between the immune checkpoint PD-L1 and B7-H3 activates the classic aerobic glycolytic pathway PI3K-AKT mTOR in tumor cells (65). Moreover, due to glucose deprivation and downregulation of mTOR activity, the glycolytic signaling pathway PI3K-AKT mTOR is inhibited in T cells (66). In addition, the CTLA-4 pathway competitively inhibits CD28-mediated co-stimulation and reduces Akt phosphorylation and activation, thereby impairing T-cell glucose metabolism and mitochondrial remodeling (67). Moreover, the interaction between immune checkpoints and their ligands (such as PD-1/PD-L1 and CTLA-4/CD86) further participates in the metabolic reprogramming of tumor cells and immune cells (68). In summary, the synergistic effect of tumor metabolism regulation and inhibition of immune checkpoints in the TME can activate the host anti-tumor immune response.

**Figure 3:**
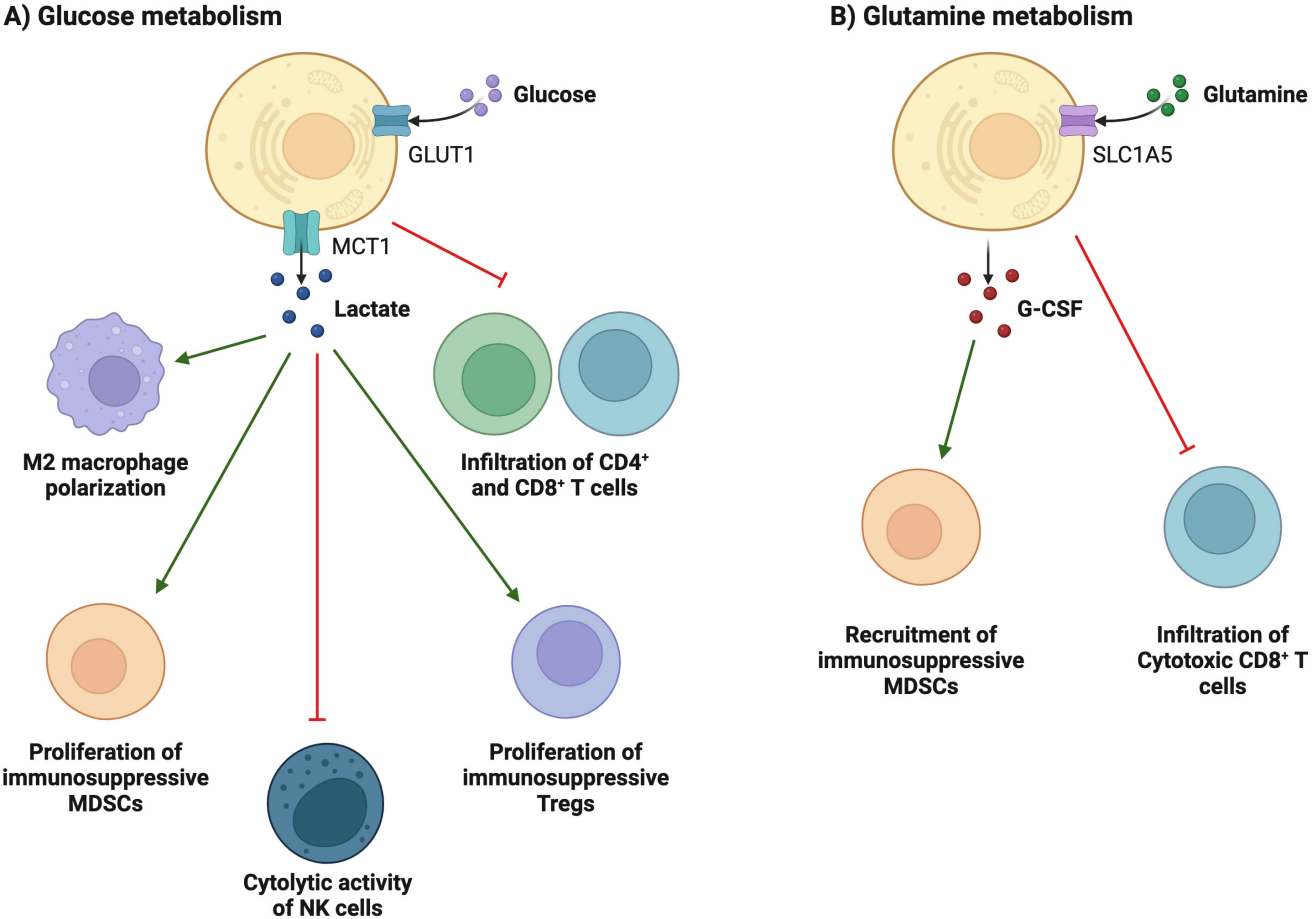
Metabolic reprogramming is associated with immunosuppression and evasion. **(A)** Tumor cells consume large amounts of glucose, which is associated with poor T cell infiltration. High glucose depletion leads to the production and secretion of lactate into the TEM, where lactate acts in an immunosuppressive manner, reduces the cytolytic activity of NK cells, and enhances PD-1 expression and the immunosuppressive capacity of Treg cells. In addition, lactate increases the frequency of MDSCs in tumors and spleen and induces "M2-like" polarization in tumor-associated macrophages. **(B)** glutamine depletion in cancer cells decreases the activation and infiltration of CD8+ T cells and enhances the recruitment of MDSCs by increasing the secretion of G-CSF.

**Figure 4:**
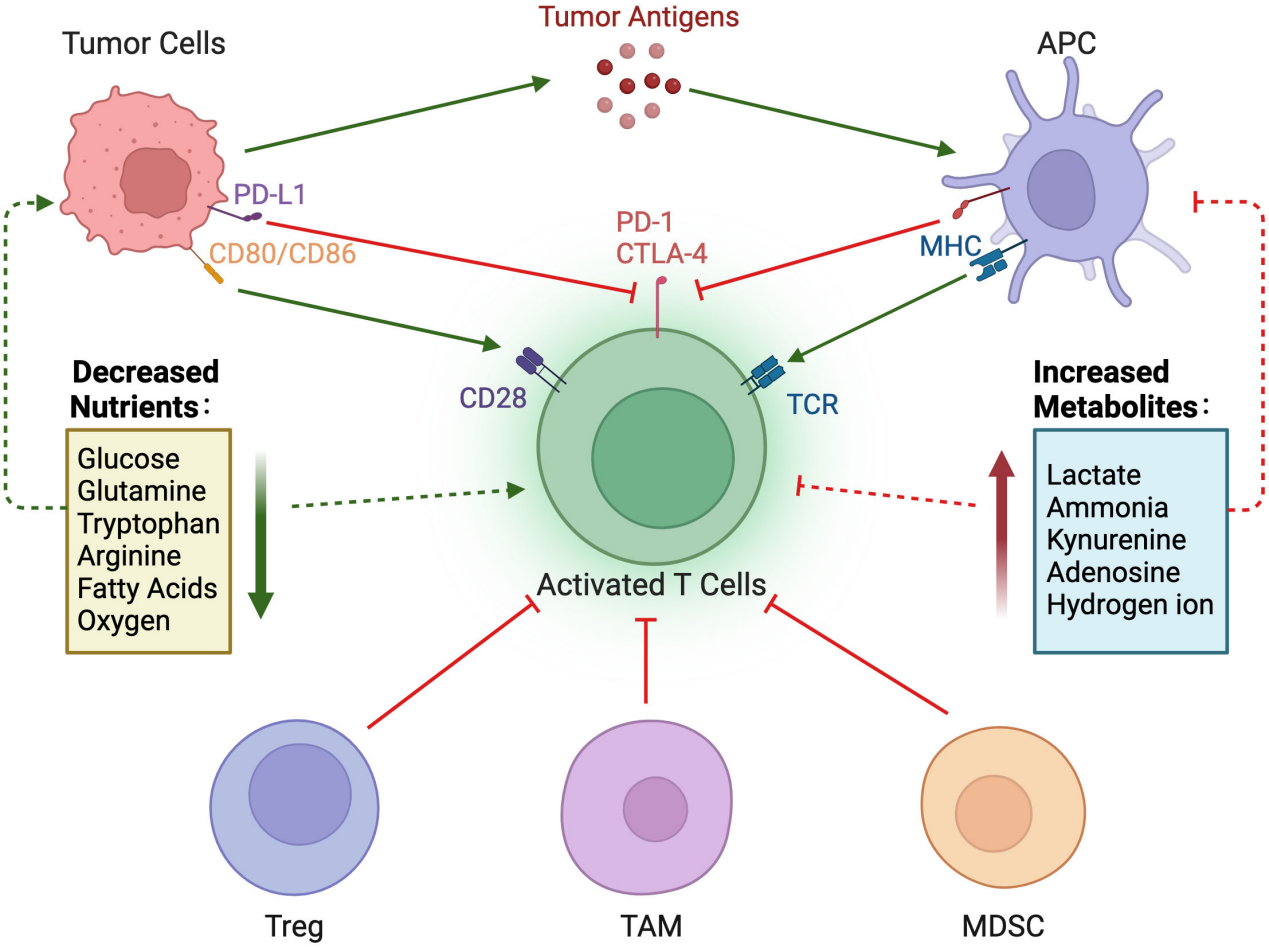
Nutrient regulation of immune responses in the tumor microenvironment. In the tumor microenvironment, a decrease in nutrients and an increase in immunosuppressive metabolites can impair the immune response to tumors.

Compared with normal cells, tumor cells utilize a large amount of glucose to produce lactate under aerobic conditions and provide the amino acids and intermediate metabolites of the pentose phosphate required for tumor cell proliferation. The accumulation of lactate in the TME has profound effects on immune cells. In **Figure 3A**, tumor cells consume large amounts of glucose, which is associated with poor CD4^+^ and CD8^+^ T cell infiltration (69). High glucose depletion leads to the production and secretion of lactate into the TME, where lactate acts in an immunosuppressive manner, reduces the cytolytic activity of natural killer (NK) cells, and enhances PD-1 expression and the immunosuppressive capacity of Treg. In addition, lactate increases the frequency of MDSC in tumors and spleen and induces “M2-like” polarization in tumor-associated macrophages. Additionally, the accumulation of lactate in tumors leads to a pH decrease and hinders the ability of dendritic cells (DC) to recognize and present antigens, as well as the stability of antigen MHC-I complexes (**Figure 4**) (20). Acidification also reduces the ability of mannan receptors (MR) to bind to antigens, inhibits glycolysis, and promotes monocyte differentiation into monocyte-derived dendritic cells. Moreover, lactate in tumors inhibits toll-like receptor 3 (TLR3) and stimulator of interferon genes (STING) leading to interferon*-γ* reduction, accelerates antigen degradation, and impairs cross-presentation. Pilon-Thomas et al. (70) reported that neutralizing the acidity of TME with bicarbonate can increase T cell infiltration and improve response to immunotherapy in immune checkpoint inhibition and adoptive cell transfer therapy.

### 4.2 Amino acid metabolism

Amino acids are essential for maintaining the rapid proliferation of tumor cells. Besides as substrates for protein synthesis, amino acids play an important role in energy production, lipid and nucleic acid synthesis, and cellular redox homeostasis maintenance. The increased demand for amino acids results in tumor cells becoming strongly dependent on exogenous amino acids or reprogramming of amino acid metabolism. Alterations in amino acid metabolism can be used not only as clinical biomarkers of cancer progression but also as therapeutic targets.

Glutamine is an important metabolic fuel that helps meet the high demand for energy, biosynthetic precursors, and reducing agents in rapidly proliferating cancer cells (71). In **Figure 2**, glutamine is transported to the cytoplasm through the solute carrier family 1, member 5 (SLC1A5) (72), and is converted to glutamate in the mitochondria through a deamination reaction catalyzed by glutaminase. Glutamate is converted to the TCA cycle intermediate α-ketoglutarate (α-KG) by glutamate dehydrogenase (73). α-KG is a critical metabolite that serves in both ATP production and in replenishing TCA cycle intermediates (74). Glutamine metabolism is essential for developing effector T cells (75). The overexpression of inhibitory receptors (such as CTLA-4 or PD-1) can inhibit the upregulation of glucose and glutamine metabolism after TCR involvement and co-stimulation (76, 77). The interaction between PD-1 and PD-L1 or PD-L2 can inhibit the PI3K-Akt mTOR pathway (78) and disrupt T cell metabolic reprogramming, including glutaminolysis (79). Huang et al. (80) reported that SLC1A5 overexpression can stimulate the growth and survival of colon cancer cells. In **Figure 3B**, glutamine depletion in cancer cells decreases the activation and infiltration of CD8^+^ T cells and enhances the recruitment of MDSC by increasing the secretion of granulocyte-colony stimulating factor (69).

Arginine is a versatile amino acid. In addition to being a building block in protein synthesis, it is a precursor of nitric oxide (NO), creatine, and polyamines. The arginine metabolic pathway is shown in **Figure 2**, and there are two main metabolic pathways: Arginine is metabolized into citrulline and NO by nitric oxide synthase, and into ornithine and urea by arginase. Ornithine is an important resource for putrescine, which is a key precursor for polyamines. NO plays a diverse role in tumorigenesis and metastasis, and the promotion or inhibition of tumors depends on sensitivity to NO, exposure time, and NO concentration (81). Low concentrations of NO (<100 nmol/L) may promote tumor development by inhibiting apoptosis and stimulating endothelial cell proliferation. In contrast, high concentrations of NO (400-1000 nmol/L) would disrupt the cell cycle and accelerate cellular apoptosis (82). Because tumor cells rely on arginine in the TME, depriving tumors of arginine has emerged as a novel anti-tumor therapy and has shown encouraging efficacy in clinical trials against certain types of cancer. However, simply depriving arginine not only starves tumor cells but also impairs the anti-tumor immune response. Geiger et al. (83) reported that the reduction of intracellular arginine negatively affected the survival of T cells. When arginine is completely depleted in the medium, the cell cycle of T cells is arrested in the G_0_-G_1_ phase. Arginine supplementation, while enhancing the anti-tumor immune response, may support tumor growth. Both deprivation and supplementation alter arginine levels in the TME, which influences all cells. To achieve tumor-specific arginine restriction without influencing immune cells, an in-depth study of differences in arginine metabolism between tumor cells and immune cells is needed.

Tryptophan, an essential amino acid, plays a vital role in protein biosynthesis and serves as a precursor for the synthesis of a variety of important bioactive compounds. As shown in **Figure 2**, tryptophan is first converted to N-formyl kynurenine, which is deformylated by arylformamidase to kynurenine (Kyn) (84). Kyn then activates the transcription factor aryl hydrocarbon receptor (AhR). Activated AhR could upregulate levels of PD-1 in T cells, inhibiting the anti-tumor immune response (85). Tryptophan metabolism and its key enzymes affect a variety of cell biology functions, including immune response, cell proliferation, and migration, through interactions with downstream molecules or pathways. Le Naour et al. (86) found that tryptophan is associated with restricting immune response in the TME and is often upregulated in human tumors. Targeted therapy based on tryptophan metabolism provides a new and potentially advantageous therapeutic strategy for cancer.

### 4.3 Lipid metabolism

Lipids are essential nutrients for cells, the main structural components of cell membranes, signal molecules, and energy providers. The most common lipids include fatty acids, triglycerides, sphingolipids, phospholipids, and cholesterol (87). In addition, abnormal fatty acid metabolism is related to the occurrence and development of tumors, such as liver cancer, gastric cancer, ovarian cancer, and cervical cancer (88–90).

The sterol regulatory element binding protein pathway in lipid metabolism controls lipid synthesis in cells (91). In **Figure 2**, *De novo* synthesis of lipids can promote high expression of PD-L1 and tumor immune suppression through palmitoylation of the immune checkpoint PD-L1 (92). In addition, PD-1 stimulates the AMP-activated protein kinase activity, inhibits glycolysis, and promotes fatty acid oxidation by upregulating activated CD4^+^ T cells, thereby inhibiting the development of effector T cells (93). The lipid transporter protein CD36 mediates intracellular fatty acid uptake and lipid droplet growth (94). CD36-mediated fatty acid uptake in tumor-infiltrating CD8^+^ T cells activates lipid peroxidation and iron apoptosis, while CD36 deficiency inhibits tumor growth (95). In addition, CD36 on the surface of CD8^+^ TILs takes up oxidized low-density lipoprotein and induces lipid peroxidation, thereby promoting CD8^+^ T cell dysfunction in tumors (96). Cholesterol metabolism regulates the antitumor activity of CD8^+^ T cells. Inhibition of acetyl-CoA acetyltransferase-1 increases plasma membrane cholesterol levels and enhances the effector and proliferation function of CD8^+^ cells (97).

## 5 Metabolic modulation of humoral immunity

The phenotype and function of T cells in the TME have been extensively studied, but the role of immune metabolism in B cell function, differentiation, and its impact on tumor immunity remains elusive, perhaps partially due to their range of actions and heterogeneity. B cell activation triggered by antigen binding to the B cell receptor (BCR) triggers naive B cell proliferation and differentiation into plasma cells that produce specific antibodies. This process requires increased energetic activity to supply sufficient energy and raw material. Upon receiving antigenic stimulation, the metabolic requirements of B cells undergo significant changes. Activated B cells rapidly increase glycolysis, the TCA cycle, and oxidative metabolism, whereas naive B cells exhibit metabolic quiescence (98). Upon activation of the BCR, B cells tend to aerobic glycolysis and produce more lactate. Antigen-stimulated B cells undergo glycolysis mainly before the S phase. Once in the S phase, B cells skew to the pentose phosphate pathway, possibly to provide ribose-5-phosphate for nucleotide synthesis and NADPH for redox homeostasis (99). Additionally, plasma cells utilize amino acid metabolism, mitochondrial respiration, and the TCA cycle to maintain antibody secretion (100). Glycolysis is increased in regulatory B cells and their differentiation is associated with hypoxic environments, but their metabolic profile remains incompletely understood (101).

In the TME, B lymphocytes can exhibit anti-tumor or pro-tumor characteristics depending on localization, tumor type, TME, and antibody isotype (102). For example, B lymphocytes in hepatocellular carcinoma can express anti-tumor cytokines, e.g., IFN-γ, IL-12, TRAIL, and granzyme B (103). In contrast, B cells can also secrete anti-inflammatory molecules, e.g., TGF-β, IL-10, and IL-35, and release immunosuppressive factors, such as PD-L1, Tim-1, and FASL (104–106).

In B-cell lymphoma, the redox state of tumor cells is imbalanced due to rapid proliferation. To avoid cell damage and apoptosis caused by abnormal oxidative stress, the pentose phosphate pathway is selectively activated by serine/threonine protein phosphatase type-2A to produce NADPH to maintain redox homeostasis. There are “metabolic checkpoints” during the development of B cells to avoid overactivation and malignant transformation. For example, the transcription factors paired box 5 and Ikaros family zinc finger 1 in B cells can limit intracellular energy metabolism. This process reduces glucose uptake and ATP synthesis, limiting cell proliferation by restricting energy metabolism (107). The use of agonists of paired box 5 and Ikaros family zinc finger 1 increases the expression of glucose feedback sensor thioredoxin interacting protein and cannabinoid receptor 2 restricting the glucose uptake and limiting the B cells glycolysis, in combination with glucocorticoids, and has a potential therapeutic effect in B-cell lymphoma. Additionally, Feist et al. (108) reported that the aspartate aminotransferase glutamic-oxaloacetic transaminase 2/signal transducer and activator of transcription-3/p65 signaling pathway is essential for lymphocyte malignant proliferation. Xiong et al. (109) reported that MYC-phosphate cytidylyltransferase 1 choline-α induced abnormal choline metabolism and hindered necrosis in B-cell lymphoma. These studies reveal the potential clinical application prospects of using exogenous metabolic modulators to limit the proliferation of malignant tumors. In summary, tumor metabolism affects tumor therapy by altering the TME and resetting immune cells. Tumor metabolomics is becoming a new research hotspot.

## 6 Advanced artificial intelligence methods in tumor metabolomics

Tumor metabolism and the TME are complex, dynamic systems that play pivotal roles in tumor progression and immune evasion. Traditional methods for studying tumor metabolism, such as chemical screening, gene knockout, and mass spectrometry imaging, have provided valuable insights but also face limitations in terms of operational complexity and personalized metabolic analysis (110). To overcome these challenges, AI has emerged as a powerful tool, revolutionizing the field of metabolomics by enabling more robust and comprehensive data analysis.

**Figure 5:**
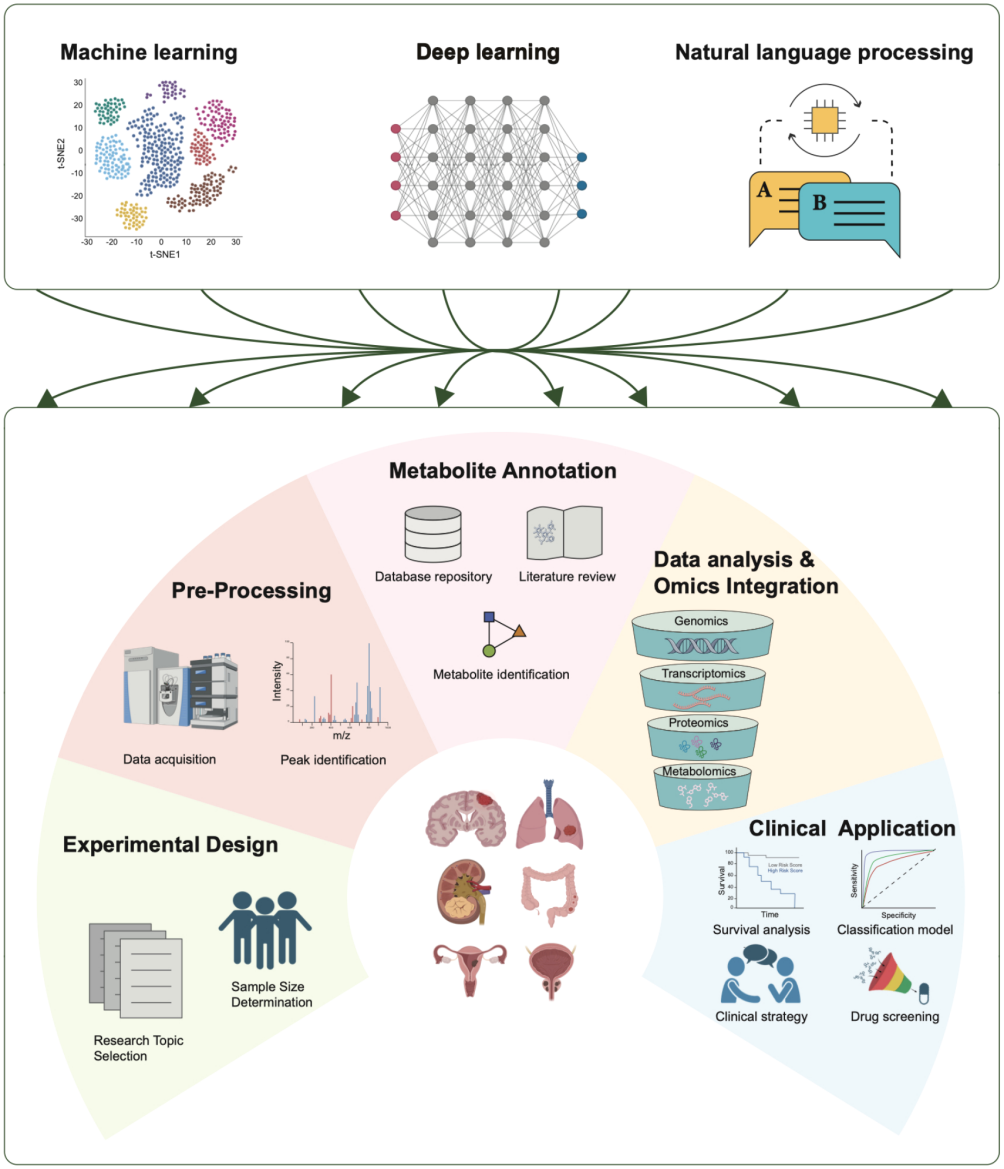
Applications of AI in tumor metabolomics. AI technologies play crucial roles in various stages of tumor metabolomics research, including experimental design, data preprocessing, metabolite annotation, data analysis and omics integration, and clinical applications. Three AI technologies—machine learning, deep learning, and natural language processing—contribute significantly to each stage.

The study of tumor metabolism requires the integration of various omics technologies, including metabolomics, epigenomics, and proteomics, with a primary focus on metabolomics. Metabolomics is a technique used to analyze small molecule compounds and can be divided into four categories based on research objectives: metabolite target analysis, metabolite fingerprint analysis, metabolite profile analysis, and metabolomics analysis. With advancements in sequencing technology and AI, researchers are developing more robust methods for metabolite analysis and quantification via data-driven approaches (**Figure 5**) (111).

AI encompasses a range of tools and mathematical methods that enable computer systems to perform tasks and make decisions that typically require human intelligence. AI technologies can be classified into several main categories. Unsupervised learning involves modeling the underlying structure of unlabeled data via techniques such as principal component analysis (PCA) (112), t-SNE (113), and autoencoders (112). On the other hand, supervised learning learns from labeled datasets to focus on predictive tasks. Deep learning, which includes multilayered neural networks such as artificial neural networks (ANNs) (112), automatically learns hierarchical features from raw data and has greatly supported metabolomics research. Additionally, natural language processing (NLP) models such as BERT and GPT have introduced new approaches to metabolomics research (114, 115). These AI advancements aid biologists and medical researchers in various aspects of omics studies, including experimental design, data preprocessing, metabolite annotation, data analysis, multi-omics integration, and clinical applications. This study summarizes the main steps in metabolomics research and the development of AI technologies (**Table 1**).

**Table 1:** Available ML algorithms of metabolomics.

Tool/Algorithm Name	Data Processing Stage	Omics Type	Functionality	Method Category	Specific algorithm	Publication Year	Reference
	Sample collection & Pre-Processing	NMR	Spectrum Reconstruction & Peak Intensity Calibration	ML/DL	LSTM, DNN	2019	(116)
	Sample collection & Pre-Processing	GC-MS	Peak Detection, Peak Classification	ML/DL	CNN	2019	(117)
konly	Sample collection & Pre-Processing	LC-MS	Peak Detection, Peak Integration	ML/DL	CNN	2020	(111)
ChromAlignNet	Sample collection & Pre-Processing	GC-MS	Peak Alignment, Peak Filtering	ML/DL	LSTM (Long Short-Term Memory)	2019	(118)
	Sample collection & Pre-Processing	LC-MS	Peak Detection, Artifact Elimination	ML/DL	CNN	2019	(119)
	Metabolite Annotation	Raman spectra	Component Identification	ML/DL	CNN	2018	(120)
	Metabolite Annotation	FTIR, MS	Structure Identification, Functional Group Prediction	ML/DL	Autoencoder, MLP (Multi-Layer Perceptron)	2020	(121)
	Metabolite Annotation	LC-MS	Spectral Prediction, Fragmentation Prediction	ML/DL	ANN	2016	(122)
NEIMS	Metabolite Annotation	GC-MS	Spectral Prediction	ML/DL	MLP	2019	(123)
DeepCCS	Metabolite Annotation	IMS	CCS Value Prediction	ML/DL	CNN	2019	(124)
DarkChem	Metabolite Annotation	MS	m/z and CCS Value Prediction, Structure Prediction	ML/DL	VAE (Variational Autoencoder)	2020	(125)
MSHub	Sample collection & Pre-Processing	GC-MS	Auto-deconvolution, Fragmentation Pattern Analysis, Data Sharing, Molecular Networking,m/z and Retention Time Shift Analysis	ML/DL	One-Layer Neural Network	2021	(126)
Combat	Data Analysis/Omics Integration	MS	Batch Correction	ML/DL	Empirical Bayes Framework	2007	(127)
MNN	Data Analysis/Omics Integration	MS	Batch Correction	ML/DL	Mutual Nearest Neighbor (MNN)	2018	(128)
Spec2Vec	Data Analysis/Omics Integration	LC-MS	Spectral Similarity Calculation, Library Matching, Molecular Networking	LLM/NLP	Word2Vec	2021	(129)
METLIN	Metabolite Annotation	LC-MS	Compound Identification	ML/DL	Input-Output Kernel Regression Model	2018	(130)
MassBank	Metabolite Annotation	GC-MS	Compound Identification, Data Repository	ML/DL	Weighted Cosine Correlation	2010	(131)
SIRIUS 4	Metabolite Annotation	LC-MS	Isotope Pattern Scoring, Fragmentation Tree Computation, Element Detection, Structure Prediction	ML/DL	DNN/Bayesian Network Scoring	2019	(132)
CANOPUS	Metabolite Annotation	LC-MS	Compound Class Annotation	ML/DL	DNN (Deep Neural Network)	2020	(133)
MS2Mol	Metabolite Annotation	LC-MS	*De Novo* Structure Prediction, Unknown Metabolite Identification	LLM/NLP	Transformer	2023	(134)
Mmvec	Data Analysis/Omics Integration	–	Microbe-Metabolite Association	LLM/NLP	Word2Vec	2013	(135)
MetGem	Metabolite Annotation	LC-MS	Molecular Network Visualization, Spectral Similarity Calculation	ML/DL	t-SNE	2018	(113)
iMet	Metabolite Annotation	LC-MS	Structural Annotation, Novel Metabolite Identification	ML/DL	Radan forest	2017	(136)
MassGenie	Metabolite Annotation	LC-MS	*De Novo* Structure Prediction, Molecular Identification	LLM/NLP	Transformer	2021	(114)
MIST (Metabolite Inference with Spectrum Transformers)	Metabolite Annotation	LC-MS	Small Molecule Structure Elucidation	LLM/NLP	Transformer	2022	(137)
RT-Transformer	Metabolite Annotation	LC-MS	Metabolite Identification	LLM/NLP	Transformer	2024	(138)
BERT-m7G	Data Analysis/Omics Integration	–	Identification of RNA Modifications	LLM/NLP	Transformer	2021	(139)
Bert2Ome	Data Analysis/Omics Integration	–	Identification of RNA Modifications	LLM/NLP	Transformer/CNN	2023	(140)
IDSL_MINT	Metabolite Annotation	LC-MS	Annotation of Untargeted Metabolomics and Exposomics Data	LLM/NLP	Transformer/CNN	2024	(141)
MSNovelist	Metabolite Annotation	LC-MS	*De Novo* Structure Prediction, Small Molecule Structure Elucidation,Fingerprint Prediction	ML/DL	MLP	2022	(142)
MS2Prop	Metabolite Annotation	LC-MS	Property Prediction, Compound Identification	LLM/NLP	Transformer/CNN	2022	(143)
Mass2SMILES	Metabolite Annotation	LC-MS	Chemical Structure Prediction, Functional Group Prediction, Molecular Formula Prediction	LLM/NLP	Transformer/CNN	2023	(144)
MS2DeepScore	Metabolite Annotation	LC-MS	Structural Similarity Prediction, Spectral Similarity Calculation, Spectral Library Matching	ML/DL	Siamese neural network	2021	(145)

### 6.1 Data preprocessing

After acquiring mass spectrometry data, several processing steps are typically performed before the data can be annotated. These steps include format conversion, peak detection, peak alignment, calculating peak areas, and filtering to eliminate redundancies (111, 146).

Several deep learning algorithms are particularly suitable for preprocessing mass spectrometry data. For example, convolutional neural networks (CNNs) excel at capturing local features by detecting patterns such as peaks and characteristic signals through convolution operations (43, 112, 147). Furthermore, CNNs achieve scale invariance through pooling operations, enabling the model to recognize the same features at different scales, thus enhancing robustness and generalization (148). Melnikov et al. (111) developed the “peakonly” algorithm, utilizing CNNs to enhance peak detection and integration in LC-MS data for metabolomics, achieving high precision and flexibility in managing noisy peaks. Similarly, research by Risum A.B (117)., Kantz (119), and Lim (120) demonstrates the application of CNNs for tasks such as peak identification and alignment. Improved RNN structures, like long short-term memory (LSTM) networks and gated recurrent units (GRUs), can capture long-range dependencies, aiding in analyzing temporal correlations in mass spectrometry data (149). For instance, Li and colleagues developed ChromAlignNet based on LSTM, a deep learning model that significantly improves peak alignment in GC-MS data, outperforming existing methods and requiring no user input for reference chromatograms and parameters (118). Metabolomics spectral and mass spectrometry data, essentially continuous two-dimensional data, can be encoded for processing by large language models. However, there currently lacks a dedicated natural language processing model for this preprocessing task, which warrants attention.

### 6.2 Metabolite annotation

Metabolite annotation involves translating spectral patterns into matching chemical molecules. The most common approach involves matching mass spectra (GC-MS’s electron ionization (EI) and LC-MS’s tandem MS) with reference library spectra of known molecules (150). This step is currently the most widely applied machine learning application in metabolomics, as AI can enhance both matching and reference library generation.

The main steps in spectral matching involve comparing the query spectrum with the library spectrum and calculating similarity scores. Machine learning models, such as the DNN-based SIRIUS 4 (132), the MLP-based MSNovelist (142), and the CNN-based DeepCCs (124), are widely used for spectral prediction, fragmentation prediction, and structure identification. Notably, large language models (LLMs), a type of deep learning AI model, excel in various tasks, particularly natural language processing (NLP) (151). LLMs, such as OpenAI’s GPT-X and Google’s BERT, are composed of neural networks with numerous parameters and trained on vast amounts of unlabeled data via self-supervised or semi-supervised learning (151). These models are also used in protein structure prediction and drug screening (151).

LLMs’ capabilities in handling complex data, pattern recognition, and reading scientific literature make them suitable for spectral matching and metabolite annotation. For example, Fakouri Baygi et al. (141) developed IDSL_MINT, a cheminformatics deep learning framework based on transformer models, which predicts molecular fingerprint descriptors from MS/MS fragmentation spectra and facilitates the training of predictive models from various mass spectral libraries. RT-Transformer is another deep neural network model that uses graph attention networks and 1D-Transformer to predict retention times via various chromatographic methods, showing competitive performance and excellent scalability, thus increasing the accuracy of metabolite identification in liquid chromatography (138).

Although still in the early exploration stages and limited by the number of tokens in semantic libraries, LLMs present a promising solution for handling larger databases and various forms of metabolomics annotation sources.

### 6.3 Multi-omics joint analysis

We are in an era of data explosion, where the advancement of sequencing technologies has accumulated an unimaginable volume of data over the past few decades. This includes genomics, transcriptomics, epigenomics, proteomics, and microbiomics. Several successful examples have demonstrated the powerful effect of integrating multi-omics in biological and medical research. For instance, Chakraborty et al. (152) integrated ChIP-Seq and RNA-Seq data to study head and neck squamous cell carcinoma (HNSCC), finding that tumor-specific histones H3K4me3 and H3K27ac are associated with transcriptional changes in HNSCC driver genes, such as EGFR, FGFR1, and FOXA1. Additionally, Vaske et al. (153) used the path recognition algorithm (PARADIGM) integrated with genomic model data to infer the activity of patient-specific biological pathways from multi-omics data, better identifying changes in tumor-related metabolic pathways in glioblastoma multiforme (GBM) and breast cancer datasets. Mo et al. (154) hypothesized that different molecular phenotypes can be predicted through a set of orthogonal latent variables, which represent different molecular driving factors. They proposed a new framework that uses generalized linear regression to construct a joint model of categorical and numerical variables (both continuous and discrete) from integrated genomics, epigenomics, and transcriptomics data (154). In another study, integrating metabolomics and transcriptomics revealed molecular perturbations underlying prostate cancer. The metabolite sphingosine demonstrated high specificity and sensitivity for distinguishing prostate cancer from benign prostatic hyperplasia. Downstream of sphingosine, impaired sphingosine-1-phosphate receptor 2 signaling represents a loss of tumor suppressor function and a potential key oncogenic pathway for therapeutic targeting (155).

The combined effect of multi-omics analysis exceeds the sum of its parts, thus necessitating the integration and joint analysis of various omics data. However, different omics data have distinct analysis workflows and information densities. For example, transcriptomics data provide gene expression levels characterized by their quantitative nature, while proteomics and metabolomics data are obtained through mass spectrometry, which is more complex and diverse (156).

This integration presents several challenges, requiring robust computational methods to handle these challenges and effectively extract inter-omics relationships from heterogeneous, large-scale, and noisy biological data generated across different platforms, technologies, tissues, and species (149, 156). AI is a promising approach to address these challenges.

In metabolomics, AI technologies can be applied in multiple areas, including data preprocessing, feature extraction, pattern recognition, and data integration. For instance, deep learning algorithms can identify feature peaks in mass spectrometry data, enhancing the accuracy of metabolite identification (111, 117, 149). Additionally, machine learning methods can be used for the integrative analysis of multi-omics data, helping to uncover complex biological networks and pathways (156).

The analysis of metabolomic data and the integration of multi-omics typically involve steps such as correcting batch effects, inferring networks, reducing data dimensionality, and recognizing patterns. Batch effects, which result from differences in experimental conditions and personnel, can mask true biological variations, thereby impacting the accuracy and reliability of analyses (157).

AI is especially suited for exploring complex patterns and interpreting non-linear effects within such data. AI techniques, by learning the hidden structures and relationships in large, high-dimensional datasets, help researchers uncover complex biological signals that might be overlooked by traditional statistical methods (151). This capability is crucial for effectively integrating diverse omics data, providing a comprehensive view of biological systems. ComBat, an algorithm operating within an empirical Bayesian framework, is commonly used for batch correction (127). It adjusts batch effects by estimating and modifying batch-specific parameters to align them closer to pooled estimates, harmonizing data across different batches while preserving biological signals. Another sophisticated method for batch correction is the Mutual Nearest Neighbors (MNN) technique (128), which uses shared nearest neighbor information to correct batch discrepancies. By aligning nearest neighbors across batches, MNN effectively harmonizes datasets.

In terms of data correlation network, MMvec, inspired by word2vec, uses matrix factorization to analyze the co-occurrence patterns of microbes and metabolites, offering insights such as the metabolites produced by microbes in specific diseases (135). Similarly, Similarity Network Fusion (SNF) integrates multiple omics datasets by creating separate networks for each data type and merging them using non-linear fusion techniques based on message-passing theory (158). This integration deepens as the networks converge through successive iterations. Network-Based Multi-group Data Integration (NetICS) provides a strategy for integrating diverse group data in tumor gene sequencing (159). It utilizes network diffusion models on directed functional interaction networks to predict the impacts of genetic, epigenetic, and miRNA variations on downstream genes and proteins.

Multi-omics datasets encompass a wide range of data types and sizes, from gene or metabolite abundances in hundreds of samples to additional dimensions like sample origin and clinical data in single-cell studies, posing the ‘curse of dimensionality’ (160). Moreover, the high correlation among variables can introduce multicollinearity, complicating the identification of significant biological markers. Specific machine learning methods for metabolomics, such as PCA (112), t-SNE (161), and NMF (162), facilitate data reduction and visualization. However, deep learning and large language models surpass these methods by offering superior capabilities for feature learning and contextual understanding, thus expanding potential applications in complex biological data analysis.

### 6.4 Biomarker research

Metabolomics not only provides crucial insights into metabolic pathways and physiological states but also plays a significant role in disease diagnosis, drug development, and nutritional research. However, the development of biomarkers faces considerable challenges, as traditional methods are time-consuming, costly, and often lack accuracy (163, 164). The rapid advancement of AI technologies, particularly machine learning and deep learning, has significantly increased the efficiency and accuracy of biomarker development. Machine learning and deep learning algorithms, such as convolutional neural networks (CNNs), recurrent neural networks (RNNs) (112), and ANNs (112), excel in handling complex and high-dimensional data, capturing patterns and relationships that traditional methods might overlook. Compared with conventional models, the incorporation of these technologies has improved the predictive capabilities of biomarkers.

For instance, in a study on gastric cancer diagnosis and prognosis prediction, Chen et al. (165) utilized a machine learning random forest model to analyze plasma samples from 702 participants across multiple centers. They developed a diagnostic model comprising ten metabolites, achieving a sensitivity of 0.905, significantly surpassing the sensitivity of traditional cancer protein marker methods (less than 0.40) (165). Similarly, in a study by Kuwabara et al. (166) liquid chromatography-mass spectrometry (LC-MS) and the alternative decision tree (ADTree) algorithm were used to analyze 2602 saliva samples for colorectal cancer detection. Their model achieved an AUC value of 0.870 in distinguishing colorectal cancer from healthy controls, whereas traditional detection methods typically have AUC values around 0.70 (166).

The methods for establishing predictive and diagnostic models in metabolomics primarily include traditional machine learning algorithms and deep learning algorithms (167, 168). Among traditional machine learning algorithms, logistic regression (LR) is widely used for binary classification problems due to its simplicity, ease of interpretation, and fast computation (169). It has shown good performance in disease prediction based on metabolites. For example, in a study on endometrial cancer (EC) by Bahado-Singh et al. (170) a logistic regression model was developed using multiple metabolites and demographic characteristics. This model combined C14:2, phosphatidylcholine with acyl-alkyl residue sum C38:1 (PCae C38:1), and 3-hydroxybutyric acid, achieving an AUC (95% CI) of 0.826 (0.706-0.946), with a sensitivity of 82.6% and specificity of 70.8% (170). However, logistic regression is less capable of modeling complex non-linear relationships and may underperform when handling high-dimensional data with complex interactions.

Random forest (RF), on the other hand, improves model robustness and resistance to overfitting by constructing multiple decision trees and averaging their results, making it suitable for high-dimensional data processing. For instance, in a study on colorectal cancer by Telleria et al., RF and logistic regression models were used to develop an accurate predictive model based on several metabolites. This model combined hemoglobin (Hgb), bilirubin E,E, lactosyl-N-palmitoyl-sphingosine, glycocholenate sulfate, and STLVT, achieving an accuracy of 91.67% (95% CI 0.7753-0.9825), with a sensitivity of 0.7 and specificity of 1 (171).

Support vector machine (SVM) constructs hyperplanes for classification, making it suitable for small samples and high-dimensional data with good generalization ability. In a study on breast cancer by An et al., an SVM model was developed based on 47 metabolites. This model achieved high accuracy in breast cancer prediction (AUC = 1), with an AUC of 0.794 for breast cancer *vs* healthy controls (HC), and 0.879 for benign *vs* HC in the testing cohort (172).

Bayesian methods, based on Bayes’ theorem, can handle uncertainty in data and are suitable for small sample learning, although they require high computational complexity and rely on prior information. In a study on early lung cancer by Xie et al., a Naive Bayes algorithm was used to develop a predictive model based on six plasma metabolites. This model significantly distinguished early lung cancer patients from healthy individuals, achieving an AUC of 0.989, with a sensitivity of 98.1% and specificity of 100.0%. The study also identified the top five important metabolites as potential biomarkers for early lung cancer screening (173).

CNN uses combinations of convolutional layers, pooling layers, and fully connected layers to extract spatial features, making them suitable for image and time-series data processing. In metabolomics research, CNNs have demonstrated excellent feature extraction and prediction performance, although they require large amounts of data and high computational resources. In a study on cholangiocarcinoma and pancreatic adenocarcinoma by Urman et al., a neural network (NN) algorithm was used to develop predictive models based on multiple lipid and protein biomarkers. This model differentiated between benign strictures and cholangiocarcinoma patients with an AUC of 0.984, a sensitivity of 94.1%, and a specificity of 92.3%. The same method was also used to distinguish pancreatic adenocarcinoma patients from control groups, achieving an AUC of 0.98, a sensitivity of 88%, and a specificity of 100% (174). Furthermore, the study evaluated the performance of other machine-learning algorithms. The authors used a Bayesian variant of the general linear model (BGLM) and the C5.0 decision tree algorithm on the same data. Both C5.0 and BGLM demonstrated good performance in feature selection and prediction, although their predictive power was slightly lower than that of the NN algorithm.

ANN simulates the structure of human brain neurons and learns complex features and patterns through multiple layers of neurons and weight adjustments (116). ANN has strong feature extraction capabilities and is suitable for processing large-scale data, but it requires extensive data for training and high computational resources, and the model interpretability is poor. In a study on oral cancer by Monedeiro et al., an ANN model was developed based on nine relevant volatile organic compounds (VOCs). These compounds included 1-octen-3-ol, hexanoic acid, E-2-octenal, heptanoic acid, octanoic acid, E-2-nonenal, nonanoic acid, 2,4-decadienal, and 9-undecenoic acid. The model’s performance was assessed using 10-fold cross-validation and receiver operating characteristic curves, achieving an overall accuracy of 90%, with 100% sensitivity and specificity for oral cancer cases (175).

In **Table 2**, we summarize the recent applications of different AI technologies in establishing predictive or diagnostic models through metabolomics. Various predictive models have achieved very high performance in their respective cohorts. It is important to note that most studies have employed cross-validation with separated cohorts and lack independent external validation cohorts. Most cohorts consist of around a hundred samples, which may lead to overfitting when using AI models to build predictive models, potentially resulting in poor validation performance in real-world external cohorts (167). The main reasons for the lack of external validation cohorts are data acquisition difficulties and high costs. Establishing comprehensive and interconnected channels and large integrated databases through AI technology may be a feasible approach in the future.

**Table 2:** AI-based metabolomics studies for disease prediction and diagnosis.

Disease Category	Discovery Cohort Size	Validation Cohort Size	Metabolomics Technique	Feature Count	Feature Types	Modeling Method	AUC Value	Sensitivity	Specificity	Reference
Cancer cachexia	192	–	NMR	15	Mixed Metabs	RF, LR	0.991	0.9464	–	(176)
Endometrial cancer	116	47	NMR	181	Mixed Metabs	LR	0.826	0.826	0.708	(170)
Colorectal cancer	130	344	Targeted Metabolomics	3	Amino Acids	LR	0.955	0.968	0.833	(170)
Colorectal cancer	60	60	UPLC-MS/MS	25	Mixed Metabs	RF, LR	0.917	0.7	1	(171)
Breast cancer	114	75	NMR	3	Lipoprotein	LASSO	0.999, 0.892	–	–	(177)
Breast cancer	75	–	LC-MS	19	Lipids	SVM	0.946	0.954	0.916	(178)
Colorectal cancer	82	79	UHPLC-HRMS	10	Lipids	OPLS-DA, SVM	0.94	–	–	(179)
Gastric cardia adenocarcinoma	276	588	UPLC-MS/MS	25	Mixed Metabs	RF, LR, SVM, Cox regression	0.869-0.900	–	–	(180)
Rectal cancer	106	–	UHPLC-QTOF-MS	8	Mixed Metabs	ANOVA, PLS-DA	0.54-0.67	–	–	(181)
Lung cancer	131	–	LC-MS	241	Mixed Metabs	LR	0.886-0.934	–	–	(182)
Colorectal adenomas, Colorectal cancer	71	81	LC-MS/MS	8	Proteins	LR	–	53%-66%	0.95	(182)
Hepatocellular carcinoma, chronic liver disease	110	–	LC-MS	12	Mixed Metabs	RF	0.84	0.848	0.924	(183)
Lung cancer	110	43	LC-MS/MS	6	Mixed Metabs	NB	0.989	0.981	1	(173)
Non-small cell lung cancer	360	201	UPLC-MS/MS	8	Mixed Metabs	RF	0.981 (diagnosis), 0.954 (efficacy)	–	–	(184)
Breast cancer	282	–	SELDI-TOF-MS	3	Proteins	SVM	–	0.9645	0.9487	(185)
Breast cancer	91	20 (healthy controls)	LC-MS	1269	Mixed Metabs	RF	1	1	1	(186)
Primary central nervous system lymphoma	68	34	UHPLC-MS/MS	14	Amino Acids	LR	0.83	–	–	(187)
Colorectal cancer	514	–	LC-MS/MS	5	Mixed Metabs	LR	0.89, 0.90	–	–	(188)
Gastric cancer	72	29	LC-TOF-MS	8	Lipids	SVM	0.915	0.69	–	(189)
Non-small cell lung cancer	112	123	SELDI-TOF-MS	3	Proteins	SVM	–	0.9656	0.9479	(190)
Intrahepatic Cholangiocarcinoma	100	100	LC-MS/MS	10	Lipids	LR	0.993	–	–	(191)
Gastric cancer	181	66	UPLC-QTOF-MS	6	Bacterial Genera	RF	0.85	–	–	(192)
Chronic Pancreatitis	160	502	GC-MS	8	Mixed Metabs	NB	0.85	–	–	(193)
Non-small cell lung cancer	250	250	UHPLC-QTOF-MS	24	Mixed Metabs	LR	0.91, 0.94	–	–	(194)
Oral Squamous Cell Carcinoma	68	–	GC-MS	3	Mixed Metabs	RF	0.91	–	–	(195)
Pancreatic Cancer	55	16	GC-MS/MS	16	Mixed Metabs	LR	–	0.907	0.895	(196)
Non-Small Cell Lung Cancer	251	103	LC-MS/MS	7	Mixed Metabs	LR	0.912	–	–	(197)
Breast Cancer	113	99	LC-IT-MS	31	Nucleosides	SVM	–	0.8767	0.899	(198)
Breast Cancer	55	55	UHPLC/Q-TOF-MS	16	Mixed Metabs	LASSO	0.973	–	–	(199)
Cervical Cancer, CIN	69	–	UPLC-QTOF-MS	28	Mixed Metabs	LR	>0.80	–	–	(200)
Maternal Pregnancy Smoking	894	–	High-resolution metabolomics (HMR)	7	Cotinine and Hydroxycotinine	LR	0.81	–	–	(201)
Prostate cancer	247	139	GC-MS	22538	Mixed Metabs	LR	0.99	–	–	(202)
Pancreatic cancer	82	82	LC-MS	3	Mixed Metabs	LASSO	0.784	–	–	(203)
Papillary Thyroid Carcinoma	108	116	SELDI-TOF-MS	3	Proteins	SVM	–	0.9515	0.9397	(204)
Thyroid Nodules	78	–	Untargeted Metabolomics	15	Mixed Metabs	DNN	0.945	–	–	(205)
Oral, Breast, Pancreatic cancer	215	–	CE-TOF-MS	57	Mixed Metabs	LR	0.865 (oral), 0.973 (breast), 0.993 (pancreatic)	–	–	(206)
Rectal cancer	106	–	LC-MS	57	Mixed Metabs	PLS-DA, LR	0.88, 0.81, 0.84	0.88, 0.81, 0.84	–	(207)
Type 2 diabetes, Coronary artery disease	1538	2521	LC-MS	111	Mixed Metabs	PLS-DA, LR	–	0.73, 0.70	–	(208)
Colorectal cancer	282	291	GC-MS	29	Mixed Metabs	LR	0.996	0.993	0.938	(209)
Lung cancer	51	–	SPME-GC-MS	19	Mixed Metabs	RF	0.94	0.885	–	(210)

NB, naive bayes; LASSO, LASSO regression; LR, logistic regression; SVM, sSupport vector machine; RF, random forest.

## 7 Discussion

This review highlights the current state and strategies for applying AI algorithms to tumor metabolism studies. By exploring metabolic differences between tumor and normal cells, particularly from the perspectives of metabolomics and TME interactions, this study demonstrates the significant advantages of AI in data preprocessing, feature extraction, pattern recognition, and data integration. These technologies not only enhance the efficiency and accuracy of data analysis but also offer new perspectives for personalized medicine and precision therapy.

Compared to existing studies, this review emphasizes the unique advantages of LLMs in tumor metabolism research. LLMs can handle complex data, identify patterns, and utilize their strengths in natural language processing to improve metabolite annotation and multi-omics integration (151). This innovation provides a more comprehensive understanding and higher analytical precision in tumor research.

Despite the significant potential of AI technology in tumor metabolism research, there are some limitations. First, the training of AI models relies on large-scale, high-quality datasets, but the acquisition and standardization of such data remain challenging (151). Second, the complexity and black-box nature of AI algorithms may lead to difficulties in interpreting results, necessitating further algorithmic improvements and transparency. Additionally, the complexity and heterogeneity of the TME remain a research challenge, requiring more effective simulation and study of this complex system (211).

To address these limitations, future research should focus on several key areas. Firstly, further refinement of AI algorithms, especially in improving data quality and sample size, is essential to enhance their reliability in practical applications. Secondly, broadening the application of AI in the integration of multi-omics data will allow for the exploration of synergistic effects between different omics data and the revelation of more complex biological networks. Thirdly, deepening the study of the interaction between the TME and metabolism, using AI technology to simulate complex biological systems, will advance tumor research toward more refined and personalized directions.

The application of AI in tumor metabolism research discussed in this review holds significant theoretical importance and shows great promise in practical applications. AI technology can significantly improve the efficiency of tumor diagnosis and treatment, particularly in personalized and precision medicine (212). With continuous advancements in AI technology, future breakthroughs in clinical applications will further drive the development of biomedical research.
